# Supplementary material for: Incidence and risk factors for major bleeding among patients undergoing percutaneous coronary intervention: Findings from the Norwegian Coronary Stent Trial (NORSTENT)
Source: PLoS One. 2021 Mar 4;16(3):e0247358. doi: 10.1371/journal.pone.0247358 (PMC7932162; doi:10.1371/journal.pone.0247358)
Supplement: S1 Appendix — (DOCX) [file pone.0247358.s001.docx]

S1 Appendix

Supplement to “Incidence and risk factors for major bleeding among patients undergoing percutaneous coronary intervention: findings from the Norwegian Coronary Stent Trial (NORSTENT)” (Samuelsen PJ et al.).


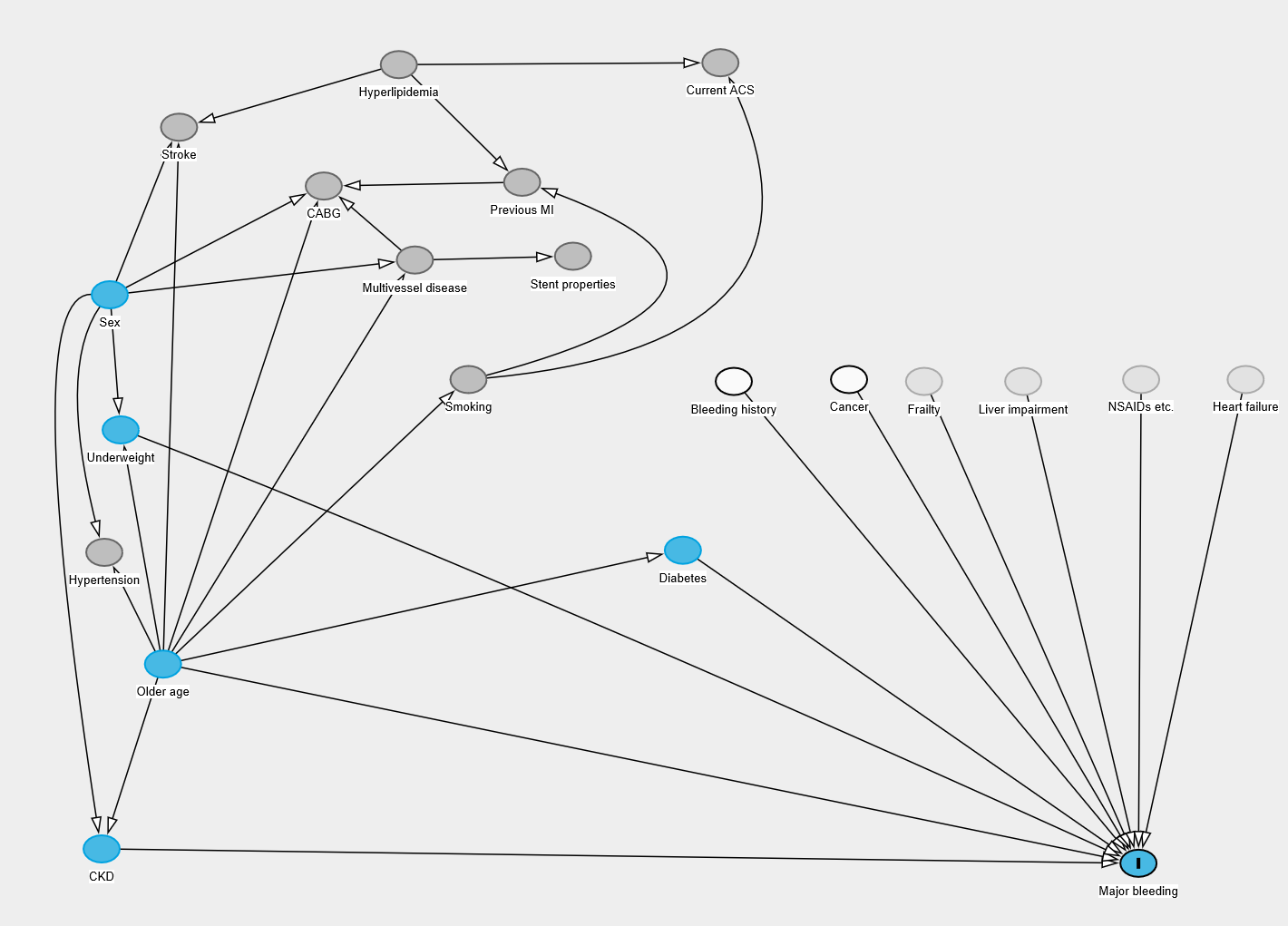


**S1 Figure:** Directed acyclic graph of the putative association between the explanatory variables and major bleeding. Low bodyweight, diabetes, older age, and chronic kidney disease are assumed to be independent risk factors of major bleeding. Variables in light gray are unmeasured. Variables in dark gray are not associated with major bleeding. Variables in white are controlled for by design, i.e., exclusion criteria.


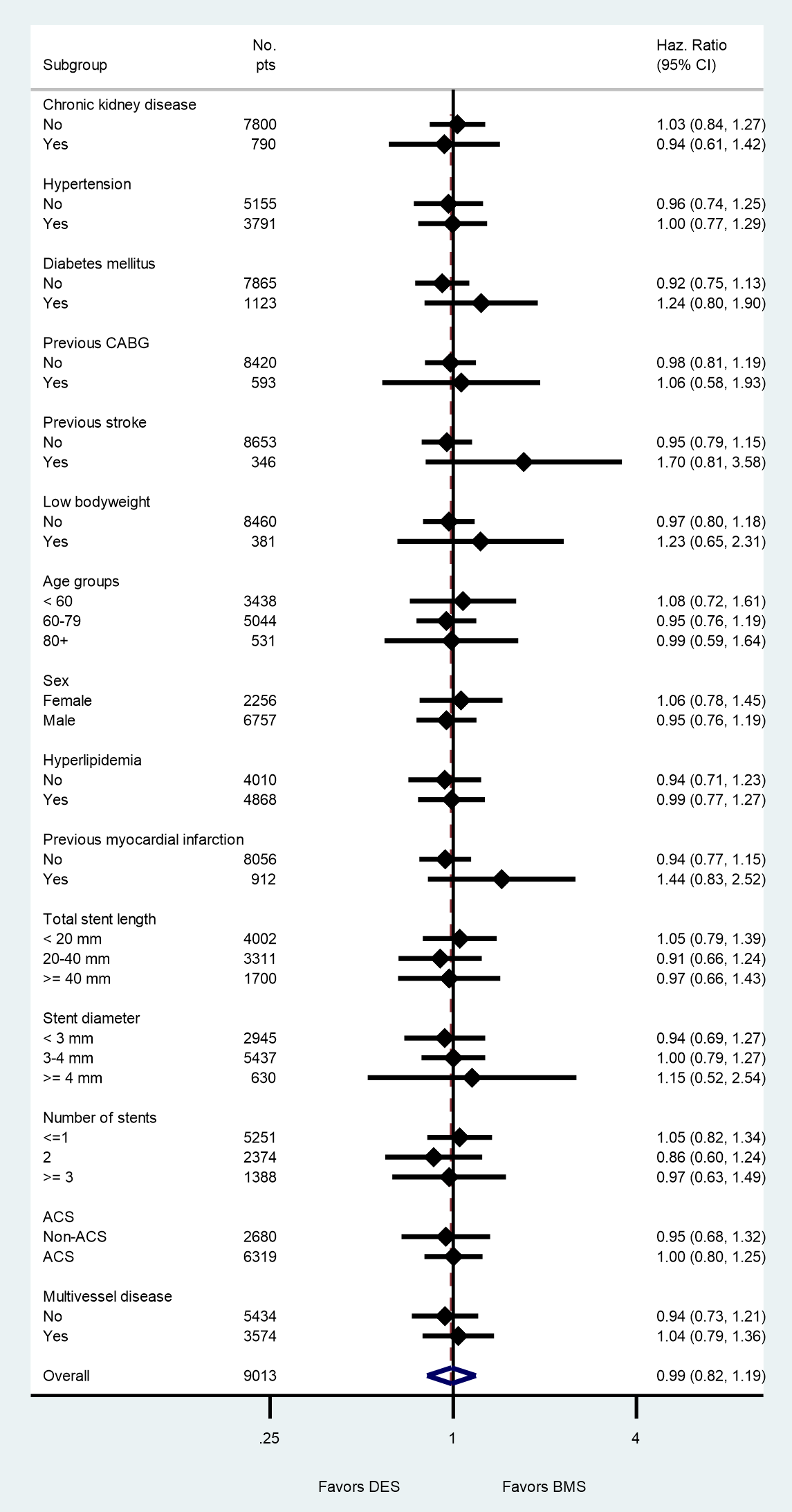


**S2 Figure:** Risk of major bleeding among those randomized to bare metal stents (BMS) compared to drug eluting stents (DES) in different patient sub groups. CABG = coronary bypass graft, ACS = acute coronary syndrome. Available case analysis.

**S1 Table:** Hazard ratios for major bleeding in the mutually adjusted model, stratified by sex. The dataset has been imputed to *N* = 9,013 by multiple imputation.

|  |  | Female | | Male | |
| --- | --- | --- | --- | --- | --- |
|  |  | HR | 95% CI | HR | 95% CI |
| CKD | No CKD | 1 | Ref | 1 | Ref |
|  | CKD | **2.21** | **(**1.50-3.26) | **1.48** | (1.04-2.12) |
| Diabetes | No diabetes | 1 | Ref | 1 | Ref |
|  | Diabetes | **1.81** | (1.22-2.69) | **1.43** | (1.04-1.96) |
| Weight | ≥ 60 kg | 1 | Ref | 1 | Ref |
|  | < 60 kg | **2.02** | (1.36-3.01) | 1.12 | (0.41-3.05) |
| Age | < 60 | 1 | Ref | 1 | Ref |
|  | 60-79 | **2.01** | (1.22-3.32) | **1.92** | (1.45-2.54) |
|  | 80+ | 1.84 | (0.92-3.70) | **4.20** | (2.71-6.51) |
| Mutually adjusted as in Table 3. For simplicity, only the main explanatory variables are reported; the other variables were statistically non-significant.  CKD: chronic kidney disease | | | | | |

**S2 Table:** Descriptive statistics of patients at baseline, stratified on those who did and did not have a myocardial infarction or percutaneous coronary intervention during follow-up.

|  |  | No MI or PCI (*n* = 7,237) | | | MI or PCI (*n* = 1,776)^a^ | | |
| --- | --- | --- | --- | --- | --- | --- | --- |
| Variable |  | *n* | *%* | *95% CI* | *n* | *%* | *95% CI* |
| Age | < 60 | 2787 | 38.5 | 37.4-39.6 | 651 | 36.7 | 34.4-38.9 |
|  | 60-79 | 4056 | 56.0 | 54.9-57.2 | 988 | 55.6 | 53.3-57.9 |
|  | 80+ | 394 | 5.4 | 4.9-6.0 | 137 | 7.7 | 6.6-9.1 |
|  |  |  |  |  |  |  |  |
| Sex | Male | 5436 | 75.1 | 74.1-76.1 | 1321 | 74.4 | 72.3-76.4 |
|  |  |  |  |  |  |  |  |
| Low bodyweight (< 60 kg) | - | 295 | 4.2 | 3.7-4.6 | 86 | 4.9 | 4.0-6.0 |
|  |  |  |  |  |  |  |  |
| Chronic kidney disease | *-* | 580 | 8.4 | 7.8-9.1 | 210 | 12.4 | 10.9-14.0 |
|  |  |  |  |  |  |  |  |
| Diabetes mellitus | *-* | 846 | 11.7 | 11.0-12.5 | 277 | 15.6 | 14.0-17.4 |
|  |  |  |  |  |  |  |  |
| Hypertension | *-* | 2967 | 41.3 | 40.2-42.4 | 824 | 46.8 | 44.4-49.1 |
|  |  |  |  |  |  |  |  |
| Hyperlipidemia | *-* | 3839 | 53.9 | 52.7-55.0 | 1029 | 58.8 | 56.5-61.1 |
|  |  |  |  |  |  |  |  |
| Current, daily smoking | *-* | 2531 | 38.2 | 37.0-39.4 | 616 | 37.9 | 35.6-40.3 |
|  |  |  |  |  |  |  |  |
| Prior CABG | *-* | 370 | 5.1 | 4.6-5.6 | 223 | 12.6 | 11.1-14.2 |
|  |  |  |  |  |  |  |  |
| Prior stroke | *-* | 260 | 3.6 | 3.2-4.1 | 86 | 4.9 | 3.9-6.0 |
|  |  |  |  |  |  |  |  |
| Prior MI | *-* | 662 | 9.2 | 8.5-9.9 | 250 | 14.2 | 12.6-15.9 |
|  |  |  |  |  |  |  |  |
| Current ACS | *-* | 5096 | 70.5 | 69.5-71.6 | 1223 | 68.9 | 66.7-71.1 |
|  |  |  |  |  |  |  |  |
| Multivessel disease | - | 2664 | 36.8 | 35.7-37.9 | 922 | 51.9 | 49.6-54.2 |
|  |  |  |  |  |  |  |  |
| Stent length | < 20 mm^b^ | 3325 | 45.9 | 44.8-47.1 | 677 | 38.1 | 35.9-40.4 |
|  | 20-40 mm | 2660 | 36.8 | 35.7-37.9 | 651 | 36.7 | 34.4-38.9 |
|  | ≥ 40 mm | 1252 | 17.3 | 16.4-18.8 | 448 | 25.2 | 23.3-27.3 |
|  |  |  |  |  |  |  |  |
| Stent diameter^c^ | < 3 mm^b^ | 2319 | 33.0 | 31.0-33.1 | 626 | 35.2 | 33.1-37.5 |
|  | 3-4 mm | 4386 | 60.6 | 59.5-61.7 | 1051 | 59.2 | 56.9-61.4 |
|  | ≥ 4 mm | 531 | 7.3 | 6.8-8.0 | 99 | 5.6 | 4.6-6.7 |
|  |  |  |  |  |  |  |  |
| Number of stents | 1^b^ | 4354 | 60.2 | 59.0-61.3 | 897 | 50.5 | 48.2-52..84 |
|  | 2 | 1884 | 26.0 | 25.0-27.1 | 490 | 27.6 | 25.6-29.7 |
|  | ≥ 3 | 999 | 13.8 | 13.0-14.6 | 389 | 21.9 | 20.0-23.9 |
| ^a^ 12.6% (*n* = 223) occurred at the index date but after the index procedure  ^b^ 70 patients did not receive a stent after randomization and are included in this group.  ^c^ 1 missing observation.  ACS: acute coronary syndrome, CABG: coronary artery bypass graft, CKD: chronic kidney disease, MI: myocardial infarction, PCI: percutaneous coronary intevention | | | | | | | |

**S3 Table:** Hazard ratios for major bleeding among patients having a new myocardial infarction or percutaneous coronary intevention in unadjusted and mutually adjusted models. The dataset has been imputed to *N* = 9,013 by multiple imputation.

|  |  | Unadjusted |  | Adjusted^a^ |
| --- | --- | --- | --- | --- |
|  | HR | 95% CI | HR | 95% CI |
| No new MI or PCI | 1 | Ref | 1 | Ref |
| Recurrent MI or PCI | **1.86** | (1.45-2.40) | **1.67** | (1.29-2.15) |
| ^a^Adjusted for chronic kidney disease, hypertension, diabetes, prior coronary artery bypass graft, prior stroke, low bodyweight, age, sex, hyperlipidemia, prior myocardial infarction, stent length, stent diameter, stent number, current acute coronary syndrome, multivessel disease, smoking, and PCI hospital | | | | |

**S4 Table:** Adherence to antiplatelet drugs. Number of daily users of antiplatelet drugs according to interview of respondents, and indicated duration at baseline.

|  | 6 months | 1 year | 2 years | 3 years | 4 years | 5 years |
| --- | --- | --- | --- | --- | --- | --- |
| Acteylsalisylic acid (ASA), *n* | 7748 | 7409 | 6904 | 6592 | 6226 | 5834 |
| Clopidogrel, *n* | 6973 | 1934 | 900 | 707 | 590 | 513 |
| Dual antiplatelet therapy (ASA + clopidogrel), *n* | 6721 | 1813 | 797 | 600 | 485 | 411 |
| Warfarin, *n* | 285 | 292 | 334 | 356 | 357 | 344 |
| Respondents, *n*^a^ | 8125 | 7836 | 7411 | 7146 | 6837 | 6544 |
| Proportion of users of DAPT with clopidogrel, % | 83 | 23 | 11 | 8 | 7 | 6 |
|  |  |  |  |  |  |  |
|  | *n* | Median | Range | IQR |  |  |
| Indicated DAPT duration at baseline (months) | 8616 | 9 | 0-24^b^ | 9-10 |  |  |
|  |  |  |  |  |  |  |
| ^a^ Respondents = those with a recorded date of interview  ^b^ Six and 24 months represent the 1^st^ and 99^th^ percentile, respectively. | | |  |  |  |  |
| Note: Users of prasugrel or ticagrelor not recorded | | |  |  |  |  |
| DAPT = dual antiplatelet therapy |  |  |  |  |  |  |
