# Supplementary material for: Incidence and risk factors for major bleeding among patients undergoing percutaneous coronary intervention: Findings from the Norwegian Coronary Stent Trial (NORSTENT)
Source: PLoS One. 2021 Mar 4;16(3):e0247358. doi: 10.1371/journal.pone.0247358 (PMC7932162; doi:10.1371/journal.pone.0247358)
Supplement: Attachment — Submitted filename: Response to Reviewers.docx [file pone.0247358.s002.docx]

# Introduction

We thank the academic editor and the reviewers for their valuable time, expertise, and attention and for providing useful comments, insights and ideas to our manuscript. We believe that those comments have increased the quality of our manuscript, and we hope we have managed to reply in a satisfactory manner. Our replies are placed directly below each specific comment. A few ideas and suggestions received from other sources during the revision stage are also addressed at the end. Changes to the manuscript are highlighted with reference to line numbers in the revised version with text mark-up. Revisions due to compliance with PLOS One’s instructions for authors are also shown in the text mark-up version.

# Technical Comments

We note that you have included the phrase “data not shown” in your manuscript. Unfortunately, this does not meet our data sharing requirements. PLOS does not permit references to inaccessible data.

**Reply:** The sentence with reference to inaccessible data has been deleted.

Thank you for including your ethics statement:  'NORSTENT (ClinicalTrials.gov: NCT0081177) has been approved by the Regional Ethics Committee
118 (REK Nord 40/2008)'.

 (a) Please amend your current ethics statement to include the full name of the ethics committee/institutional review board(s) that approved your specific study.

 (b) Once you have amended this/these statement(s) in the Methods section of the manuscript, please add the same text to the “Ethics Statement” field of the submission form (via “Edit Submission”).

**Reply:** We have amended the statement as requested.

Please ensure you have included the correct clinicaltrials.gov registration number for the clinical trial referenced in the manuscript. Currently, the clinicialtrials.gov provided in the manuscript is NCT0081177, whereas the correct registration numbers appears to be NCT00811772.

**Reply:** This is now corrected.

# Editor Comments

The authors report original results on predictors of bleeding events in the large NORSTENT cohort.
Data analysis shows no impact by the type of stent implantes (DES vs BMS) on bleeding. This is an important confirmation of previous results in a large study cohort that fit in the lane of previous evidence. A recent nationwide report (Giannini F. et al. Italian Multicenter Registry of Bare Metal Stent Use in Modern Percutaneous Coronary Intervention Era (AMARCORD): A multicenter observational study. Catheter Cardiovasc Interv. 2020 Mar 21. doi: 10.1002/ccd.28798. Online ahead of print.) showed a hyperbolic reducion in BMS use from 2013 to rates lower than 1% in 2016). This phenomenon depicts a trend that was sustanined by a growing body of evidence on the use of DES in different clinical context, including high bleeding risk patients. Please discuss this aspect.

**Reply:** The same trend is observed in the Norwegian Registry of Invasive Cardiology (NORIC) with a BMS rate below 1% in 2017 (2018 yearly report, NORIC). Previous guidelines, which were applicable in the study period in question, recommended a shorter DAPT duration after BMS implantation. Patients with high bleeding risk were therefore more likely to be given a BMS, with a corresponding shorter DAPT duration. Current European guidelines do no longer separate between BMS or DES when it comes to DAPT duration. However, for this analysis, where the participants were prescribed at least 9 months of DAPT regardless of the stent type, this becomes less of an issue. We have included a brief comment on this aspect in the discussion:

*“Since the study inclusion period (2008-11) there has been a reduction in the use of BMS; the rate of use of BMS was below one percent in 2017 in countries like Norway [24, p. 28] or Italy [25]. As the DAPT duration historically was shorter for BMS, which resulted in a reduced bleeding risk, BMS was preferentially used among patients with high bleeding risk. However, this trend did not influence the findings in this study, since the participants had similar (or close to similar) DAPT duration regardless of stent type.”* (lines: 253-9)

Comments to the Author

Reviewer #1: In this manuscript, Samuelsen investigated potential risk factors of major bleeding in the Norwegian Coronary Stent Trial. More than 9000 patients were included into the randomized control trial and followed for a median of five years. The authors observed a cumulative incidence rate of 2.3%. Additional risk factors were also identified to relate to major bleeding such as CKD, underweight, diabetes and advanced age. Overall, the study is interesting and the manuscript was well written.
Following are some comments to be considered.
• The authors merged both study arms. However, it is unclear how many samples were assigned to each study arm. It would also be useful to compare the risk factors derived from either arm.

**Reply:** We have included the sample size in each arm in the methods section; 4509 were randomized to BMS and 4504 to DES (lines: 127-8). Due to the randomization, one would not expect any differences in baseline variables between the study arms. And that is indeed the case, as previously published (Bønaa et al., 2016). In terms of major bleeding, we have already included an analysis of the potential difference in bleeding risk between BMS and DES in the different clinical subgroups, i.e., an interaction test, in the supplement. We believe that this is sufficient for this paper.

• In addition to MI, what other ischemic events were studied?

**Reply:** The ischemic events studied in NORSTENT were MI (periprocedural and spontaneous), stent thrombosis, and restenosis.

• Is there any control for medication?

**Reply:** Adherence to antiplatelet drugs were assessed through interviews at 6 months, 1 year and every following year throughout the study period. However, we did not find it correct to include this information as an adjustment variable, due to the low “resolution” of the drug data.

• The authors indicated that “the combined proportion of missing observations was 17% with a missing at random pattern”. How the missing at random pattern was assessed?

**Reply:** MAR cannot be separated from MNAR based on the data. However, we did model predictors of missingness, as described in the reference, which suggested MAR. We have included the word “possible”: “*with a possible missing at random pattern”* (line: 87)

• Is there any normalization of the study variables?

**Reply:** Normalization of variables can have different meanings, but in short: no. Continuous variables were simply converted into categorical variables, as previously used (Bønaa et al., 2016).

• How the baseline was defined for patients with multiple stent implanted? Was it counted since the last stent was implanted?
**Reply:** Staged procedures within approximately 30 days after randomization were allowed. Start-of follow up was counted from the first procedure (see full protocol in Bønaa et al., 2016).

• Is there any bleeding prior to the stent implantation?

**Reply:** Patients with prior bleeding were not excluded unless that was considered a contraindication towards long-term DAPT (560 of eligible participants were excluded due to contraindications).

• In figure 1, please list the cumulative number of events under each year of follow-up

**Reply:** The cumulative number of events are now included under each year of follow-up in Figure 1.

Reviewer #2: In the manuscript entitled "Incidence and risk factors for major bleeding among patients undergoing percutaneous coronary intervention: findings from the Norwegian Coronary Stent Trial (NORSTENT) " authors lead by Per-Jostein Samuelsen investigated the major bleeding risk factors in their cohort of 9013 patients.
This analysis shows chronic kidney disease (CKD), advanced age, and underweight as risk factors for major bleeding among patients receiving DAPT after PCI. Moreover, diabetes mellitus or recurrent MI increase bleeding risk during the follow-up.

The authors should be commended in their effort, as this analysis could support prior reports giving insights on this relevant problem. This reviewer has the following comments to raise with respect to the manuscript:

• Impact of the effective duration of DAPT is not presented in the document. Could the authors present this data, if available, and put it into perspective towards the effect of the various predictors? Is longer DAPT duration a predictor for bleeding in this analysis, could the author estimate the increase in bleeding each month of extended DAPT above 9 months?

**Reply:** Adherence to antiplatelet drugs were assessed through interviews at 6 months, 1 year and every following year throughout the study period. This data is, however, not sufficient to study the relationship between DAPT persistence and bleeding, particularly above 9 months. This question is better addressed with linkage to gold standard drug exposure data, i.e. the Norwegian Prescription Database.

We have, however, included simple descriptive statistics of the adherence to DAPT, based on those interviews, and the indicated DAPT duration (at baseline) in the supplement :

Suppl. table 1: Adherence to antiplatelet drugs. Number of daily users of antiplatelet drugs according to interview of respondents, and indicated duration at baseline.

|  | 6 months | 1 year | 2 years | 3 years | 4 years | 5 years |
| --- | --- | --- | --- | --- | --- | --- |
| Acteylsalisylic acid (ASA), n | 7748 | 7409 | 6904 | 6592 | 6226 | 5834 |
| Clopidogrel, n | 6973 | 1934 | 900 | 707 | 590 | 513 |
| Dual antiplatelet therapy (ASA + clopidogrel), n | 6721 | 1813 | 797 | 600 | 485 | 411 |
| Warfarin, n | 285 | 292 | 334 | 356 | 357 | 344 |
| Respondents, n^a^ | 8125 | 7836 | 7411 | 7146 | 6837 | 6544 |
| Proportion of users of DAPT with clopidogrel, % | 83 | 23 | 11 | 8 | 7 | 6 |
|  |  |  |  |  |  |  |
|  | *n* | Median | Range | IQR |  |  |
| Indicated DAPT duration at baseline (months) | 8616 | 9 | 0-24^b^ | 9-10 |  |  |
|  |  |  |  |  |  |  |
| ^a^ Respondents = those with a recorded date of interview  ^b^ Six and 24 months represent the 1^st^ and 99^th^ percentile, respectively. | | |  |  |  |  |
| Note: Users of prasugrel or ticagrelor not recorded | | |  |  |  |  |
| DAPT = dual antiplatelet therapy |  |  |  |  |  |  |

• PCI or MI during follow-up increased risk of bleeding. This is probably due to extending DAPT after additional procedures and subsequent peri-procedural/intrahospital events. Was this adjusted for overall DAPT duration? If this is not the case this is not interesting and the text dedicated to this result shortened.

**Reply:** For reasons explained above, we were unable to do adjustment for overall DAPT duration, including a new or extended course of treatment due to a new ischemic event. We believe that extended DAPT and procedural bleedings is a highly plausible explanation, as we already discuss in the manuscript, but we respectfully disagree that this is not interesting at all.

• BARC bleeding 3,4 or 5. Why the authors decided to also include BARC 4 in the primary endpoint ? This is hardly a marker of out-of hospital bleeding related to DAPT.

**Reply:** The endpoints were identified through screening of the National patient registry, which includes information about procedures conducted in hospitals, i.e., bleeding requiring hospital admittance. In this regard, BARC 4 is included. BARC 4 is excluded in some previous studies but not all.

• It would be meaningful to present information on the bleeding sites.

**Reply:** Agreed. However, for the current analysis, these data were unfortunately not readily available within reasonable effort**.**

• Was a treatment with clopidogrel made for all patients ? Or novel P2Y12 inhibitors have been also used ? If this is not the case this should be added in the limitation section.

**Reply:** The vast majority of patients received clopidogrel but since the study period captures the period were prasugrel was introduced, 8 patients were prescribed prasugrel at baseline. Unfortunately, we do not have data on subsequent prescribing of P2Y12 inhibitors. Preferential prescribing of ticagrelor or prasugrel to those with repeat MI or PCI, could very well explain the increased bleeding risk, as already discussed in the manuscript. This is, however, best studied with linkage to gold standard prescription registry data, and potential subject for future studies.

• Could the authors validate PRECISE-DAPT score in their population? A validation of the DAPT score would not be feasible since most patients did not do more than 9 months of DAPT.

**Reply:** Unfortunately no. We do not have baseline data on hemoglobin or white blood cell count.

• Discussion section, for the PRECISE-DAPT please report the incidence of TIMI Major or minor bleeding, comparison between BARC 3 or 5 and TIMI major bleeding only would be misleading.

**Reply:** Thank you for that clarification. We have included the number of TIMI major OR minor bleeding in the text (line: 229).

• The higher reported event rate could be also due to procedural characteristics than in 2008 -2011 were much different than today (e.g. bulkier access site, higher use of GPI). This should be reported in the study discussion and limitations.

**Reply:** We have included a comment on that in the limitations section:

*“Finally, current PCI procedural techniques have some important differences from 2008-11, including lower use of glycoprotein IIb/IIIa Inhibitors, and higher frequency of radial access site, resulting in a lower risk of bleeding [35, 36].” (line: 330-33)*

• Discussion, diabetes. "This could lead to diabetic ulcerations of the feet, and retinopathy. 257 The latter could cause intraocular bleeding compromising vision, which is classified as a BARC major 258 bleeding [8]." Could the authors support this statement with prior data from the literature. If this is not the case the conclusion here seems farfetched. In addition, the rate of intraocular bleeding is very low and hardly explain alone the association of diabetes with bleeding.

**Reply:** Upon closer consideration and review of the literature, we agree with the reviewer that this hypothesis seems farfetched. We have chosen to remove all speculations regarding possible mechanisms between diabetes mellitus and bleeding from the manuscript. Instead, we have chosen to highlight the interesting finding that an association between diabetes and bleeding have been found in studies with a predominant use of clopidogrel (and ASA) (line 276-90):

*Lemesle et al. recently reported findings from the CORONOR registry of an increased risk of bleeding in patients with stable coronary artery disease and diabetes mellitus, treated with aspirin and/or clopidogrel (roughly 40% received clopidogrel; HR = 1.75, 95% CI 1.05-2.91 among diabetic patients with HbA1C level ≥ 7%). [27]. Simek et al., on the contrary, recently report no association between diabetes mellitus and bleeding in MI patients treated with prasugrel or ticagrelor, based on information from the PRAGUE-18 trial [28]. The authors note that the bleeding risk among diabetic patients may be differential among users of clopidogrel and, particularly, prasugrel [28]. The patients included in the CORONOR, NORSTENT, and the aforementioned derivation cohort of the PRECISE-DAPT score, where an association between diabetes mellitus and bleeding has been found, mainly received clopidogrel and ASA. The exact mechanisms whereby diabetes mellitus is associated with bleeding risk is unknown. Careful interpretation is advisable while we await further data on the association between diabetes mellitus, DAPT, and bleeding risk.”*

• Abstract, row 7: the authors repeated the word “randomized” twice.

**Reply:** This is now removed.

• Line 31: please state international guidelines: also Canadian guidelines and Japanese guidelines support standardized risk scores for DAPT.

**Reply:** This is now corrected, with inclusion of references to Canadian, American, and Japanese guidelines, although our main focus is, naturally, on European guidelines (line 30-2)

• While citing Rothwell et al. wasn't the threshold used in that manuscript 70kg rather than 90 kg ?

**Reply:** Rothwell et al. report no effect of low-dose ASA in primary prevention above 70 kg. However, an increased risk of major bleeding was observed up to a body weight of 90 kg, i.e., a differential effect of body weight on efficacy and bleeding risk.

• Supplementary Figure 1 is interesting. DAG is very informative and graphic. Could the authors please explain why older age, that is independently associated to major bleeding, also provide an effect through diabetes and CKD ? is this a clinical assumption or the product of analysis ?

**Reply:** The DAG was created before the multiple regression was conducted, based on literature review, and what we believe to be plausible, i.e., clinical assumptions. However, the DAG, as presented, was also informed by simple descriptive statistics based on the actual dataset, i.e., “univariate” (simple) associations and correlations. The DAG thus explains the process for choosing the variables for the multiple regression, and possible explanations for the results (e.g., no association with stent parameters). With the final results in hand, it is perfectly reasonable to question whether there is an effect of age mediated through diabetes or CKD. We provide no answer but leave the DAG open for debate.

• Figure 2 is really informative. However, I believe that the higher rates of bleeding presented in the first block (0 to 6 months) are driven by intrahospital/periprocedural bleeding (especially considering the practice in 2008-2011 in which femoral access and GPIIb/IIIa were more extensively used). Excluding those events from the analysis would be much more informative to provide a snapshot of the bleeding risk during therapy during out-of-hospital follow-up.

**Reply:** We agree that this is partly driven by intrahospital bleeding, as can be seen from the 74 cases occurring within 30 days. However, an increased bleeding risk due to DAPT is particularly prominent in the first period after the PCI. We chose to include periprocedural bleedings in this analysis, as done in several but not all previous studies. If survival time <= 30 days (i.e., the 74 cases) is excluded, the figure will look like this:

But since we decided to include periprocedural bleeding in the first place, we will stick to the original figure.

• Table 3 could be simplified shortened eliminating the reference lines. Information about the reference groups could be included in the rows or in the footnotes.

**Reply:** Good suggestion but for maximum clarity we think we will stick with the original.

• Table 3 there is a typo for stent length >40mm (1.5q).

**Reply:** Thanks for spotting that little typo!

# A few additional changes

- We have decided to use the term “low bodyweight” instead of “underweight”, as this is more suiting particularly for women < 60 kg (while BMI < 18.5 kg/m2 is still termed underweight in the sensitivity analysis).
- Anemia is an important risk factor for bleeding. Anemia is now mentioned in the limitations section.
- Revisions due to compliance with PLOS One’s instructions for authors.
